# Editorial: Measuring progression in Multiple Sclerosis: Progressing beyond the ordinary

**DOI:** 10.3389/fnhum.2022.1095208

**Published:** 2022-11-22

**Authors:** Carynn Koch, Francesca Bagnato, Cornelia Laule, Susan A. Gauthier

**Affiliations:** ^1^Department of Neurology, Vanderbilt University Medical Center, Nashville, TN, United States; ^2^Neuroimaging Unit, Division of Neuroimmunology, Department of Neurology, Vanderbilt University Medical Center, Nashville, TN, United States; ^3^Department of Neurology, Veteran Affairs Medical Center, TN Valley Healthcare System, Nashville, TN, United States; ^4^Department of Radiology, University of British Columbia, Vancouver, BC, Canada; ^5^Pathology and Laboratory Medicine, University of British Columbia, Vancouver, BC, Canada; ^6^International Collaboration on Repair Discoveries (ICORD), University of British Columbia, Vancouver, BC, Canada; ^7^Department of Physics and Astronomy, University of British Columbia, Vancouver, BC, Canada; ^8^Department of Neurology, Weill Cornell Medicine, Cornell University, New York, NY, United States

**Keywords:** Multiple Sclerosis, progression, white matter tracts, pathophysiology, secondary progressive

Relapsing remitting Multiple Sclerosis (RRMS) makes up 85–90% of patients, with up to 75% of individuals progressing to secondary progressive MS (SPMS). While robust advances have been made in therapeutics targeting relapsing stages, much is still unknown about the pathologic mechanisms underlying the transition to progressive forms of disease. Although progression likely starts early, it becomes clinically evident only later in the disease course. The inability to detect and measure progression is a significant obstacle toward the development of medications targeting progressive disease. This Research Topic provided an overview of how progression in MS can be measured, beyond the common clinically available metrics. The contributions to this topic addressed two important perspectives on measuring progression: (1) the use of methods to predict progression through stages of disease and (2) the identification of networks that underlie common symptoms in early stages of MS.

When considering predictors of progression, not only the quantity of lesion burden but the location of lesions is known to be important. In a systematic review, Yang et al. showed brainstem and cerebellar clinically isolated syndrome have a higher risk of early conversion to a clinically definite MS; while infratentorial lesions in RRMS are not as reliable in predicting progression to SPMS. Their review summarized that while some studies have reported brainstem and cerebellar lesions may be associated with rapid progression to SPMS or even treatment failure in the second year; others have concluded that these lesions carry no significance in the timing of conversion to SPMS. These inconsistencies need further investigation, as infratentorial lesions are well-known to contribute to poor overall prognosis and higher disability rate. Along the same lines, two other Research Topic contributions put their focus on critical white matter tracts, showing that lesions in these areas tend to impact disability progression in a clinically measurable way. Yoon et al. used a combination of white matter tractography and a multi-b shells diffusion weighted MRI to indirectly quantify axonal injury in the transcallosal and corticospinal white matter tracts. They found that lesion and non-lesional related injury in transcallosal white matter tracts was more predictive of motor disability, than similar injury in corticospinal tracts. Similarly, Oladosu et al. reported the importance of pathology located in the corpus callosum, another strategic transcallosal tract, along with the optic radiations. Further, they found that in normal appearing white matter, higher density white matter bundles with small diameter and low dispersion were more susceptible to damage in people with SPMS. These studies highlight the importance of disease localization. They uphold an understanding that the differences detected in lesional and non-lesional specific white matter areas are likely to give insight into the microstructural changes that precede severe and irreversible injury, and thus possibly the transition from RRMS to SPMS.

Imaging metrics are important but ultimately progression remains intimately connected with the clinical manifestations of the disease, e.g., frequency and severity of clinical relapses and symptoms outside overt relapses. Early detection and initiation of treatment can prevent costly relapses. To this end, the remaining three Research Topic contributions focused their work on patients in the early stages of MS, with attention to how non-motor symptoms can often be challenging to quantify and contextualize in the landscape of progressive disease. It is known that MS disrupts functional networks in the brain, and in doing so causes hyperconnectivity in other networks to compensate. These compensatory mechanisms are thought to be most active in the mild stages of RRMS, often delaying clinical manifestation of disease. As an example, fatigue is one common non-motor symptom that is reported in up to 80% of people with MS. Previous studies have captured structural and functional correlates of MS, but primarily in patients with moderate to severe disability. The review by Sobczak et al. looked at fatigue in patients with mild stages of RRMS and found that elevated levels of fatigue were associated with a higher functional connectivity in the posterior salience network—a region known to play an important role in information processing. To expand on this concept, de Aratanha et al. looked at how patients in the early stages of MS activated the dorsolateral prefrontal cortex and supplementary motor area when performing a motor activity at the same time as a cognitive task, to reflect activities of daily life. They found that in easier tasks, people with MS had higher measures of unilateral cortical activity than healthy controls and in difficult tasks, showed a bilateral increase in cortical activity compared to healthy controls. This suggests that despite compensatory mechanisms in early stages of MS, it is possible to detect significant differences in cortical demand—even when these differences are not captured on the Expanded Disability Status Scale or Fatigue Severity Score.

The final contribution to this Research Topic focused on people with MS with optic neuritis, a very common first symptom. In this retrospective study, Park et al. addressed the need for improved sensitivity in testing with visual evoked potentials (VEP) and aimed to investigate whether low contrast VEP could improve sensitivity to optic neuritis with the goal of identifying optic neuritis in patients with mild or unremarkable visual impairment. This study revealed that in subclinical optic neuritis detection, VEP with low contrast stimuli detected abnormalities 53.1% of abnormalities while conventional VEP with high contrast detected 9.9% of abnormalities—making it reasonable to conclude low contrast VEP is more sensitive that the conventional high contrast VEP.

In conclusion, this Research Topic provided a comprehensive overview on the measurement of progression in MS. Using novel imaging techniques, expansion of well-established diagnostic tools, and innovative approaches to studying non-motor symptoms—these articles have highlighted how measuring progression in MS is quickly expanding beyond our ordinary tools.

## Author contributions

CK, FB, CL, and SG wrote this editorial and approved the submitted version of this editorial.

## Conflict of interest

The authors declare that the research was conducted in the absence of any commercial or financial relationships that could be construed as a potential conflict of interest.

## Publisher's note

All claims expressed in this article are solely those of the authors and do not necessarily represent those of their affiliated organizations, or those of the publisher, the editors and the reviewers. Any product that may be evaluated in this article, or claim that may be made by its manufacturer, is not guaranteed or endorsed by the publisher.

